# Prevalence and Characteristics of Acquired Coronary Fistulas After Successful Revascularization of Chronic Total Occlusion

**DOI:** 10.3389/fcvm.2021.690890

**Published:** 2021-12-22

**Authors:** Rong Fan, Haipeng Tan, Yanan Song, Wang Yao, Min Fan, Zheyong Huang, Junbo Ge

**Affiliations:** ^1^Department of Cardiology, Yueyang Hospital Integrated Traditional Chinese and Western Medicine, Shanghai University of Traditional Chinese Medicine, Shanghai, China; ^2^Department of Cardiology, Zhongshan Hospital, Fudan University, Shanghai, China

**Keywords:** acquired coronary fistulas, chronic total occlusion, percutaneous coronary intervention, coronary perforation, revascularization

## Abstract

**Background and Objectives:** Acquired coronary fistulas (ACFs) are rare coronary artery abnormalities in patients with chronic total occlusion (CTO). It has been found after revascularization, and it may cause fluster during the CTO percutaneous coronary intervention (CTO PCI). How to distinguish between ACFs and coronary perforation (CP) is very important for CTO operators. Chronic total occlusion reopening may reveal the microchannel of the adventitial vascular layers. Some of ACFs have been seen after revascularization. This study aimed to investigate the characteristics of ACFs after successful CTO PCI.

**Methods:** The clinical and procedural characteristics, medical history, and findings in electrocardiography (ECG), echocardiography, and coronary angiography were collected from 2,169 consecutive patients undergoing CTO PCI between January 2018 and December 2019 and analyzed retrospectively.

**Results:** About 1,844 (85.02%) underwent successful CTO PCI with complete revascularization. Acquired coronary fistulas were found in 49 patients (2.66%): the majority of patients with ACFs were men (81.63 vs. 60.78%; *p* = 0.016) and younger (62.8 vs. 66.69 years; *p* = 0.003), and had a history of myocardial infarction (MI) or Q-wave (69.39 vs. 54.21%; *p* = 0.035); 38 (77.55%) patients had multiple fistulas (≥3), and ACFs affected multiple branches of the CTO vessel (≥3) in 29 (59.18%) patients. None had pericardial effusion, tamponade, and hemodynamic abnormality before or after PCI.

**Conclusion:** Acquired coronary fistulas after successful CTO PCI are mainly present in young and male patients with a history of MI, and they often involve multiple fistulas and distal CTO vessels.

## Introduction

Coronary fistulas were first described as congenital and abnormal vascular connections between coronary arteries and cardiac chambers or with other vessels, but other factors may also cause coronary fistulas (1), including trauma, surgery, severe coronary atherosclerosis, and myocardial infarction (MI). Myocardial infarction is a common cause of acquired coronary fistulas (ACFs) (2, 3), and ACFs secondary to MI are usually harmless, common in patients with chronic total occlusion (CTO), and hard to identify before complete revascularization because of insufficient collateral filling. In recent years, the increasing percutaneous coronary intervention (PCI) of CTO is done successfully with new equipment and techniques, and more ACFs can be visualized after CTO percutaneous coronary intervention (CTO PCI). It is important to distinguish ACF from coronary perforation (CP), a rare but potentially serious complication in PCI, which can lead to pericardial effusion and tamponade, often necessitating medical treatment and even emergency pericardiocentesis or cardiac surgery (4). To date, the imaging characteristics, medical history, and predictors of ACFs have never been evaluated in a real-word cohort of patients who underwent successful CTO revascularization. This study was undertaken to investigate the prevalence and characteristics of ACFs after successful revascularization of CTO.

## Methods

### Patients

A total of 2,169 consecutive patients who underwent CTO PCI at ZhongShan Hospital of Fudan University between January 2018 and December 2019 were retrospectively reviewed. Patients were excluded if the PCI was undertaken in the case of acute coronary syndrome. The patients with CP were also excluded because some of them failed to complete revascularization. All original angiograms were reviewed by two physicians to confirm ACFs and to collect other clinical information. The study was approved by the Institutional Review Board (KYSKSSB2020-135).

### Procedures

All patients received antiplatelet treatment before the procedures, and unfractionated heparin was administered intravenously at 100 IU/kg followed by further addition of heparin as necessary to achieve a target activated clotting time of 250–350 s. Microcatheters include Finecross (Terumo, Japan) and Corsair (Asahi, Japan); CTO guidewires include Fielder XTR and Fielder XTA (Asahi, Japan), Gaia 2 and 3 (Asahi, Japan), Pilot 150 and 200 (Abbott, America), Conquest Pro (Asahi, Japan), Ultimate Bros 3 (Asahi, Japan), which had been used widely during the procedures.

### Study Definitions

Chronic total occlusion was defined as a complete occlusion with thrombolysis in MI (TIMI) flow grade 0 antegrade for ≥3 months (5). Procedural success was defined as <50% residual stenosis with antegrade TIMI flow grade 3 at the end of procedures (6). Acquired Coronary Fistulas was defined as an abnormal connection between coronary arteries and cardiac chambers or with other vessels, secondary to exogenous or endogenous injury. History of MI was identified by medical records, including stent thrombosis-segment elevation MI (STEMI) or non-STEMI (NSTEMI) based on initial electrocardiography (ECG) and clinical and laboratory findings. Adverse events included the death of any cause, stent thrombosis (ST)/Q-wave MI, emergent cardiac surgery, and cardiac tamponade. The coronary collaterals in CTO were classified using the Rentrop grade as follows (7): 0 = none; 1 = filling of side branches of the artery to be dilated *via* collateral channels without visualization of the epicardial segment; 2 = partial epicardial filling of the occluded artery; and 3 = complete epicardial filling of the occluded artery.

### Data Collection

Demographic, procedural, and medical data were obtained by reviewing the catheterization laboratory database and medical records of patients.

### Statistical Analysis

Continuous variables are presented as mean ± SD and were compared using Student's or the Welch's *t*-test, and one-way ANOVA followed by Dunnett's multiple comparisons tests when more than two groups were compared. Categorical variables are presented as numbers and percentages and were compared using the chi-square test or Fisher's exact test. All analyses were performed using SPSS version 26, and a value of *p* < 0.05 was considered statistically significant.

## Results

### Baseline Clinical Characteristics

Among the 2,169 consecutive patients who underwent CTO PCI, procedures were successfully conducted in 1,844 (85.01%) patients. Among them, ACFs were found in 49 (2.66%) patients: 25 (51.02%) patients had hypertension, 15 (30.61%) had diabetics, and five (10.20%) had hyperlipidemia; 23 (46.94%) patients were long-term smokers; 10 (20.41%) had a history of prior PCI, and none had previous coronary artery bypass surgery (CABG). As shown in Table 1, patients with ACFs were younger (62.8 vs. 66.69 years; *p* = 0.003), men (81.63 vs. 60.78%; *p* = 0.016), and had higher rates of MI history or Q-wave MI (69.39 vs. 54.21%; *p* = 0.035). Fielder XTR and GAIA 2 were commonly used to cross the lesion in those patients.

**Table 1:** Demographic and baseline clinical characteristics.

**Variable**	**Acquired coronary fistulas**		***p***
	**No (*n* = 1,795)**	**Yes (*n* = 49)**	
Age (years)	66.69 ± 8.12	62.8 ± 11.24	0.016^*^
Male	1,091 (60.78)	40 (81.63)	0.003^*^
Symptom			0.839
Asymptom	141	4 (8.16)	
Stable angina	901	24 (48.98)	
Unstable angina	610	17 (34.69)	
Dyspnea	84	2 (4.08)	
Syncope	59	2 (4.08)	
NYHA class			0.543
I	341	7 (14.29)	
II	888	26 (53.06)	
III	412	11 (22.45)	
IV	154	5 (10.20)	
Hypertension	994 (55.38)	25 (51.02)	0.545
Diabetes	513 (28.58)	15 (30.61)	0.756
Hyperlipidemia	363 (20.22)	5 (10.20)	0.083
Smoking	664 (36.99)	23 (46.94)	0.156
History of MI	518 (28.86)	14 (28.57)	0.965
History of MI or Q-wave MI	973(54.21)	34 (69.39)	0.035^*^
Previous PCI	474 (26.40)	10 (20.41)	0.347
Previous CABG	48 (2.67)	0	0.246
Echocardiography			
LVPWT (mm)	9.70 ± 1.43	9.63 ± 1.36	0.755
IVST (mm)	10.22 ± 1.90	10.26 ± 1.88	0.800
LVEDD (mm)	40.92 ± 8.05	41.47 ± 8.25	0.640
LVESD (mm)	53.79 ± 6.97	54.14 ± 6.69	0.757
LVEF (%)	51.07 ± 10.15	48.94 ± 9.76	0.147
Ventricular aneurysm	49(2.73)	2 (4.08)	0.569
CTO technique			0.578
Antegrade	1,570 (87.47)	44 (89.80)	
Retrograde	186 (10.36)	5 (10.20)	
Antegrade and retrograde	39 (2.17)	0	
Microcatheter			0.328
Finecross	672 (37.44)	17 (34.69)	
Corsair	1,057 (58.89)	25 (50.02)	
Finecross and corsair	66 (3.68)	0	
Guidewire for crossing occlusions			<0.001^*^
Fielder XTR	188 (10.47)	14 (28.57)	
Fielder XTA	100 (5.57)	0	
GAIA 2	409 (22.79)	20 (40.82)	
GAIA 3	268 (14.93)	5 (10.20)	
Pilot 200	258 (14.37)	10 (20.41)	
Conquest Pro	184 (10.25)	0	
Ultimate Bros 3	388 (21.62)	0	

*Data are presented as n (%), or mean ± SD. ACFs, acquired coronary fistulas; NYHA, New York Heart Association; MI, myocardial infarction; PCI, percutaneous coronary intervention; CABG, coronary artery bypass surgery; LVPWT, left ventricular posterior wall thickness; IVST, interventricular septum thickness; LVEDD, left ventricular end diastolic diameter; LVESD, left ventricular end systolic diameter; LVEF, left ventricular ejection fraction; CTO, chronic total occlusion*.

### Clinical Symptoms

The most common complaint was angina (41, 83.67%), followed by asymptom (4, 8.16%), dyspnea (2, 4.08%), and syncope (2, 4.08%). By New York Heart Association (NYHA) classification, seven patients were classified as class I, 26 as class II, 11 as class III, and five as class IV. There was no statistical difference with respect to the entire patients with CTO.

### Electrocardiography and Echocardiography

**Figure 1:**
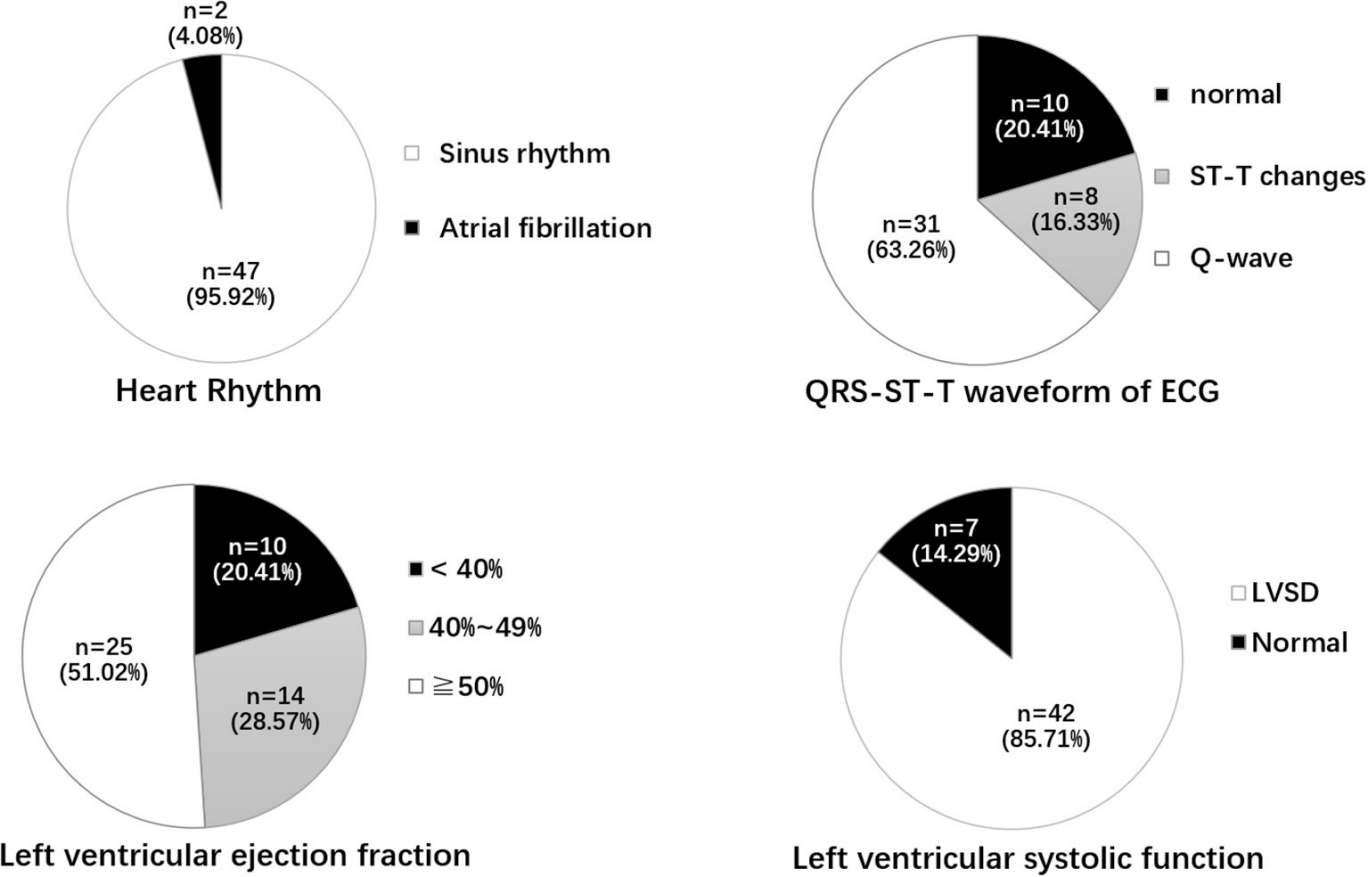
Heart rhythm, QRT-ST-T waveform of ECG, LVEF, and left ventricular systolic function. ECG, electrocardiogram; ST-T change includes depressed ST segment, Low and flat T wave; LVSD, Left ventricular systolic dysfunction; LVEF, left ventricular ejection fraction.

As shown in Figure 1, among 49 patients with ACFs, ECG displayed Q-waves in 31 (63.26%) patients, normal in 10 (20.41%) patients, and ST-T changes (depressed ST segment; low and flat T wave) in eight (16.33%) patients; 47 (95.92%) patients had sinus rhythm, and two (4.08%) had atrial fibrillation (AF).

Echocardiographic examination was performed in all the patients. The mean left ventricular ejection fraction (LVEF) was 48.94 ± 9.76%; the LVEF was <40% in 10 (20.41%) patients, 40–49% in 14 (28.57%), and ≥50% in 25 (51.02%). Forty-two (85.71%) patients had left ventricular systolic dysfunction (LVSD), and two (4.08%) had ventricular aneurysms. The mean left ventricular posterior wall thickness (LVPWT), interventricular septum thickness (IVST), left ventricular end-diastolic diameter (LVEDD), and left ventricular end-systolic diameter (LVESD) are shown in Table 1. Pericardial effusion and tamponade were not found in patients with ACFs.

### Coronary Angiography

**Figure 2:**
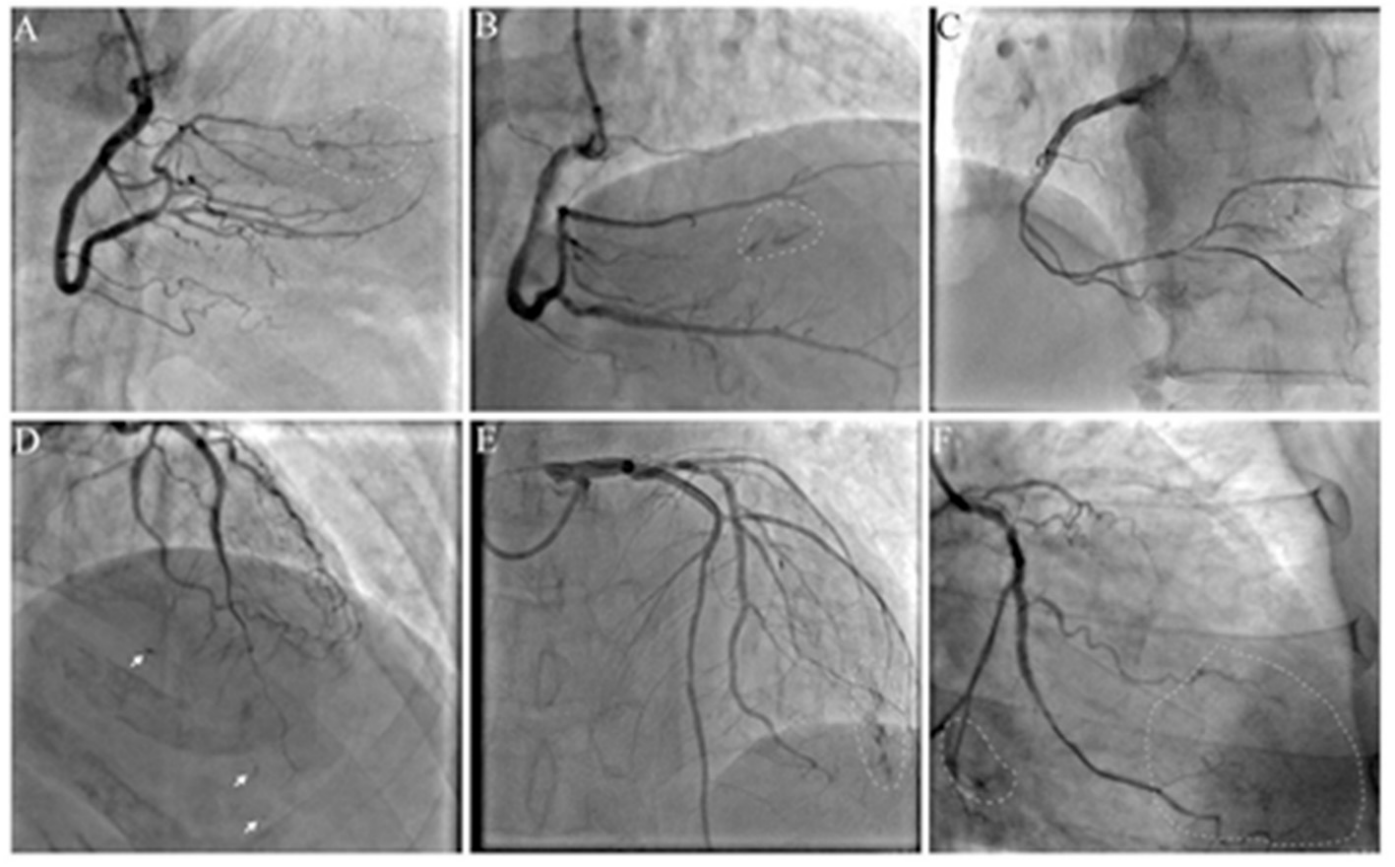
Angiographic characteristics of acquired coronary fistulas (ACFs): multiple, diffuse, and tiny. **(A–C)** ACFs were found after right coronary artery (RCA) chronic total occlusion-percutaneous coronary intervention (CTO-PCI); **(D,E)** ACFs were found after left anterior descending (LAD) CTO-PCI; **(F)** ACFs were found after left circumflex coronary artery (LCX) CTO-PCI. Arrow: spot fistulas; circle: flake fistulas; Supplementary Movies 1–6 show angiographic findings.

The angiographic characteristics of ACFs are shown in Table 2. Because more right coronary artery (RCA) CTO PCIs were performed, the most frequent origin of the ACFs was RCA (*n* = 24; 48.98%), followed by left anterior descending (LAD) (*n* = 19; 38.78%) and left circumflex coronary artery (LCX) (*n* = 6; 12.24%); 38 (77.55%) patients had multiple drainage sites (≥3), and ACFs involved multiple branches of CTO vessel (≥3) in 29 (59.18%) patients. Images of ACFs are shown in Figure 2 and Supplementary Movies 1–6. In 11 (22.45%) patients, the collateral blood supply was identified on angiography, indirectly indicating ACFs before PCI (Supplementary Movies 7, 8).

**Table 2:** Angiographic characteristics of ACFs in CTO.

**Variable**	**Acquired coronary fistulas**		***P***
	**No (*n* = 1,795)**	**Yes (*n* = 49)**	
Right coronary artery dominance	1,625 (90.53)	45 (91.84)	0.757
CTO vessel			0.952
LAD	736 (41.00)	19 (38.78)	
LCX	209 (11.64)	6 (12.24)	
RCA	850 (47.35)	24 (48.98)	
Number of ACFs holes			
<3	–	11 (22.45)	
≥3	–	38 (77.55)	
Number of affected vessel branches			
<3	–	20 (40.82)	
≥3	–	29 (59.18)	
Collateral filling of Rentrop			0.142
0	28 (1.56)	0 (0)	
1	785 (43.73)	28 (57.14)	
2	655 (36.49)	17 (34.69)	
3	327 (18.22)	4 (8.16)	
Diagnosed by collateral circulation before PCI	–	11 (22.45)	

*Data are presented as n (%). LAD, left anterior descending; LCX, left circumflex coronary artery; RCA, right coronary artery; CAG, coronary angiography*.

As shown in Table 3, clinical and imaging characteristics and medical history were similar among patients with ACF originating from RCA, LAD, and LCX, except that smoking, was more frequent in patients with ACFs originating from RCA and LAD than in those with ACFs from LCX. The mean length of hospital stay was 4.45 ± 1.99 days, and there was no death, emergent cardiac surgery, or MI during hospitalization.

**Table 3:** Clinical and angiographic characteristics of three CTO vessel subgroups.

	**LAD (*n* = 19)**	**LCX (*n* = 6)**	**RCA (*n* = 24)**	***P*-value**
Age (years)	62.21 ± 9.60	63.83 ± 12.06	63.00 ± 12.62	0.948
Male	17 (89.47)	3 (50.00)	20 (83.33)	0.089
Smoking	9 (47.37)	0	14 (58.33)	0.038^*^
Hypertension	7 (36.84)	4 (66.67)	14 (58.33)	0.268
Diabetes	6 (31.58)	2 (33.33)	7 (29.17)	0.974
Hyperlipidemia	2 (10.53)	0	3 (12.50)	0.663
Previous PCI	2 (10.53)	1 (16.67)	7 (29.17)	0.312
With clinical symptoms	16 (84.21)	6 (100)	23 (95.83)	0.284
History of MI	4 (21.05)	3 (50.00)	7 (29.17)	0.391
NYHA III–IV	4 (21.05)	3 (50.00)	9 (37.50)	0.326
Q-wave	13 (68.42)	4 (66.67)	14 (58.33)	0.779
LVEF (%)	48.63 ± 11.97	43.83 ± 11.04	50.46 ± 7.14	0.333
≥3 holes	14 (73.68)	3 (50.00)	21 (87.50)	0.126
≥3 branches vessel	11 (57.89)	3 (50.00)	15 (62.50)	0.847
Hospital stay (days)	4.79 ± 2.42	4.5 ± 1.6	4.17 ± 1.71	0.603

*Data are presented as n (%), or mean ± SD*,

* *p < 0.05*.

## Discussion

In the present study, the most common characteristics of ACFs in patients with successful CTO PCI included multiplicity and diffusibility with three or more branch vessels and drainage sites, and a history of MI or Q-wave (69.40 vs. 54.21%), which was 56.3–58.4% in the overall CTO population (8). Smoking is a risk factor for coronary artery disease (CHD) and is more common among patients with ACFs originating from LAD and RCA, which could be ascribed to the smaller sample size or lower incidence in LCX. In the presence of ACFs, the contrast agent quickly diffuses out of the coronary artery without retention during angiography.

**Figure 3:**
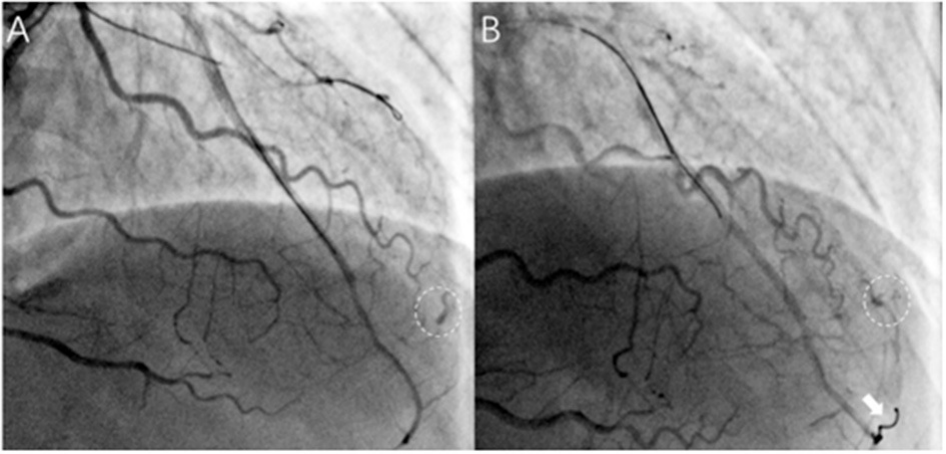
Acquired coronary fistulas uselessly and unnecessarily treated with coil embolization in a patient. **(A)** After successful recanalization of the LAD, ACFs were visualized and difficult to identify with coronary perforation timely (circle); **(B)** Rattled and successful delivery of one coil through the microcatheter (Finecross, Terumo, Japan) (arrows), but ACFs still existed (as shown in circle), and pericardial effusion was not found on echocardiography. (Supplementary Movies 9, 10 show angiographic findings).

**Figure 4:**
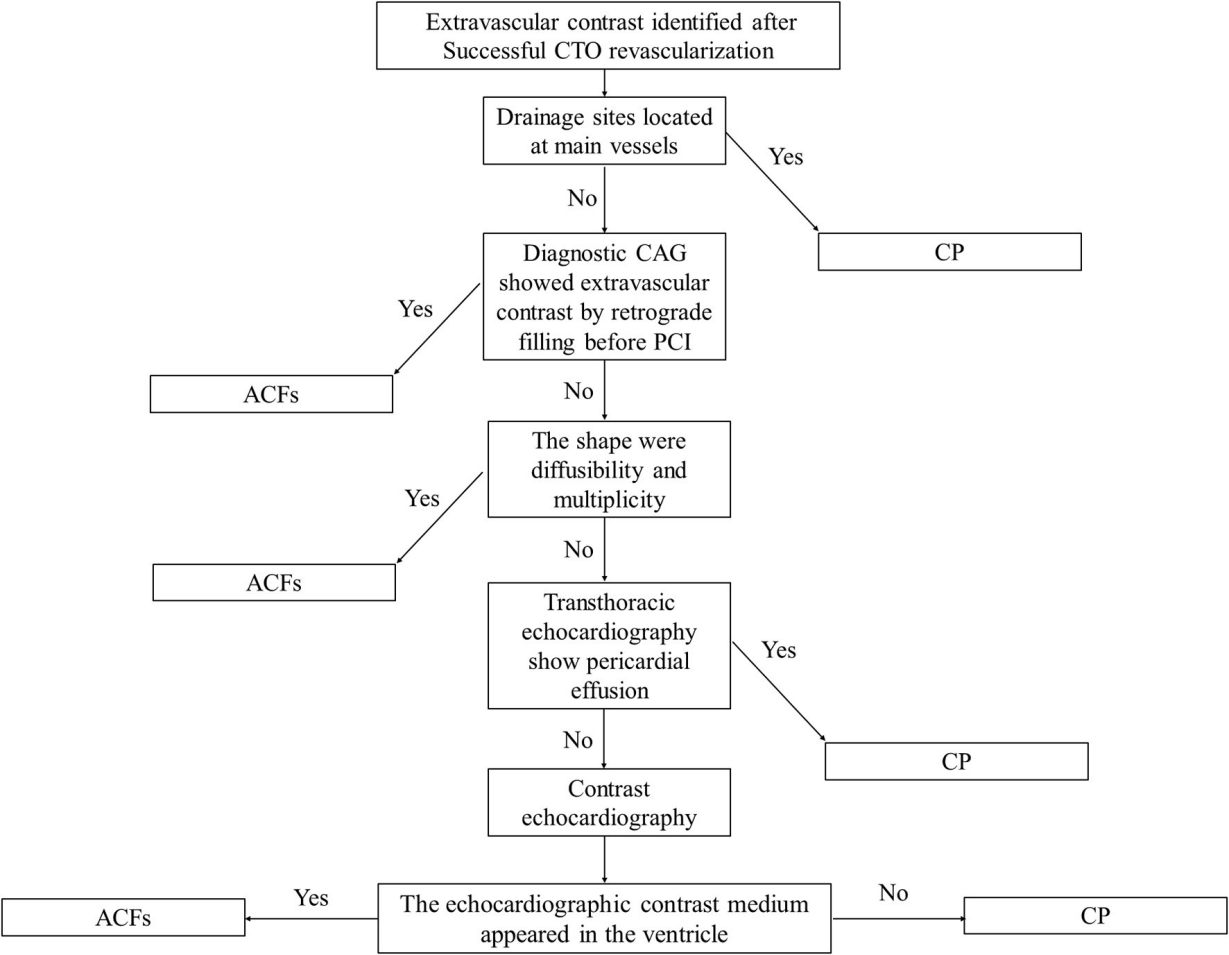
A flow chart used to distinguish between coronary perforation (CP) and ACFs after CTO reopening.

In our study, the prevalence of ACFs in the successful CTO PCI patients was 2.66%, which is lower than CP (up to 8.9%) during CTO PCI (9). On occasions, CP and ACFs have a similar angiographic appearance, and Ellis classification has been used to classify CP as type I–III (10). Unlike CP, ACFs are drained into the cardiac chambers, and therefore, pericardial effusion and tamponade do not develop, thereby rendering procedures safe. The incorrect judgment of the operator and lack of bedside echocardiography may lead to unnecessary treatment. For example, coil embolization is feasible and effective for the treatment of CP, but useless and unnecessary for ACFs (Figure 3; Supplementary Movies 9, 10). It is therefore important to distinguish ACFs from CP during CTO PCI. However, contrast echocardiography is helpful to determine whether there is a true pericardial effusion from ACFs (11). A flow chart may be helpful to distinguish between CP and ACFs after the CTO reopening (Figure 4).

Acquired coronary fistulas may result from MI, hypertrophic cardiomyopathy, dilated cardiomyopathy, tumor, PCI, CABG, cardiac transplant, cardiac biopsy, pacemaker placement, and others (12–14). Microvasculature rupture may be the most important cause of ACFs (15, 16). Sudden complete occlusion of a coronary artery may block the blood supply to the myocardium, leading to myocardial necrosis. The myocardial rupture will occur within the first 2 weeks in 90% of MI because of extensive myocardial necrosis (17, 18). Complete rupture triggers hemopericardium and sudden death, and incomplete rupture will cause the formation of a ventricular aneurysm because the pericardium closes the ventricular perforation. In the patients with CTO, in contrast, the coronary artery is obturated slowly, allowing the development of collateral circulation; myocardial necrosis does not cause symptoms and patients can survive the attack, but endocardial necrosis may cause damage to the microvasculature. Myocardial scarring and microvasculature rupture may cause ACFs, which become apparent on coronary angiography after successful CTO-PCI. Young and male patients were more likely to survive after MI attack, so our data show the ACFs were more common in them. Other underlying mechanisms for ACFs include newly developed collaterals, neovascularization of mural thrombus formation, and reopening of the Thebesian vessels. Moreover, coronary steal syndrome or hemodynamic impairment was not evident after CTO PCI in our patients with ACFs.

## Limitations

Contrast echocardiography is an ultrasound technique, which is safe and non-invasive for the assessment of drainage sites (19). This technique was not employed in the present study, and the specific location of drainage sites of ACFs was still unclear, but no pericardial effusion and tamponade were found at 2 h after PCI on transthoracic echocardiogram. Acquired coronary fistula have been reported to cause myocardial ischemia, endocarditis, and congestive heart failure (20), but long-term investigations are needed to evaluate whether ACFs are associated with poor prognosis in patients with CTO.

## Conclusion

Although ACFs are mostly benign, it is very important to recognize that ACFs may cause unnecessary treatments. The ACFs are characterized by diffusibility, multiplicity, and leakage of contrast on angiography, often found in the branches of CTO vessels, and more common in patients with a history of MI.

## Data Availability Statement

The raw data supporting the conclusions of this article will be made available by the authors, without undue reservation.

## Ethics Statement

The studies involving human participants were reviewed and approved by the Ethics Committee of Zhongshan Hospital, Fudan University. The patients/participants provided their written informed consent to participate in this study.

## Author Contributions

RF drafted the manuscript. HT collected data and conducted the statistical analysis. WY reviewed all original angiograms. YS provided the figures and their interpretation. MF, ZH, and JG helped revise the manuscript. All the authors have read and approved the final manuscript.

## Funding

This study was supported by the National Key Research and Development Program of China (No. 2016YFC1301200), the National Natural Science Foundation of China (No. 81870269), and the Foundation of Shanghai Construction of TCM Medical Service System (No. ZY[2018-2020]-FWTX-8003).

## Conflict of Interest

The authors declare that the research was conducted in the absence of any commercial or financial relationships that could be construed as a potential conflict of interest.

## Publisher's Note

All claims expressed in this article are solely those of the authors and do not necessarily represent those of their affiliated organizations, or those of the publisher, the editors and the reviewers. Any product that may be evaluated in this article, or claim that may be made by its manufacturer, is not guaranteed or endorsed by the publisher.
